# Real-World Direct Medical Costs and Clinical Outcomes of Immunotherapy and Targeted Therapy for Cutaneous Melanoma: A Bottom-Up Cost-of-Illness Study in a Public Healthcare System

**DOI:** 10.3390/cancers18213540

**Published:** 2026-11-04

**Authors:** Aleksandra M. Kovačević, Tijana G. Stanojković, Milijana N. Miljković, Viktorija M. Dragojević Simić, Bojana M. Cikota Aleksić, Nemanja K. Rančić, Lidija O. Kandolf

**Affiliations:** 1 Centre for Clinical Pharmacology, Military Medical Academy, 11000 Belgrade, Serbia; stanojkovictijana96@hotmail.com (T.G.S.); milijanamiljkovic@gmail.com (M.N.M.); vdragsim@gmail.com (V.M.D.S.); cbojana@yahoo.com (B.M.C.A.); 2 Faculty of Medicine of the Military Medical Academy, University of Defence, 11000 Belgrade, Serbia; nece84@hotmail.com (N.K.R.); lkandolfsekulovic@gmail.com (L.O.K.); 3 Institute for Radiology, Military Medical Academy, 11000 Belgrade, Serbia; 4 Clinic for Dermatovenerology, Military Medical Academy, 11000 Belgrade, Serbia

**Keywords:** cost-of-illness, direct medical costs, immune checkpoint inhibitors, melanoma, real-world evidence, Serbia, targeted therapy

## Abstract

**Background/Objectives**: Immunotherapy and targeted therapy improve melanoma survival but raise costs. Real-world cost and outcome data are scarce, especially in Central and Eastern Europe. We estimated per-patient costs and outcomes in Serbia’s public healthcare system. **Methods**: This retrospective, single-centre study assessed costs in 183 patients with cutaneous melanoma who received systemic therapy at a Serbian tertiary centre (2019–2026). Direct medical costs were reconstructed from invoices and discharge letters from the healthcare-sector perspective. Censoring-adjusted mean 24-month costs were estimated from diagnosis (*n* = 165) and treatment start (*n* = 179). Outcomes included overall survival (OS), real-world progression-free survival (rwPFS), and time to next treatment or death (TNT-D). **Results**: Mean 24-month costs per patient were EUR 66,466 (95% CI 58,465–75,334) from diagnosis (EUR 75,388 with the partitioned estimator), 94.7% for systemic therapy, and EUR 107,229 (95% CI 97,405–118,201) from treatment start. At diagnosis, costs were about twice as high in stages III–IV as in stages I–II. By stage at treatment start, costs were similar in stages III and IV (EUR 102,122 vs. 104,535) despite shorter stage IV survival (*p* < 0.001). Two-year OS was 91.5% for adjuvant and 51.9% for metastatic first-line intent. **Conclusions**: Systemic therapy drove costs, which were similar in stages III and IV despite poorer stage IV survival. The cost measurement starting point substantially affected estimates and should be reported.

## Simple Summary

Modern melanoma treatments, immunotherapy and targeted therapy have improved survival, but they are expensive, and most cost evidence comes from high-income countries. We reconstructed all documented medical resource use for 183 patients treated at a tertiary hospital in Serbia, using invoices, discharge letters, and clinical records. Over the first two years after diagnosis, the average cost per patient was about EUR 66,500, and almost 95% of it was spent on these medicines. Over two years from the start of treatment, the average cost was about EUR 107,200. Monthly costs rose almost thirtyfold once treatment began. Patients with stage III or IV disease at diagnosis had about twice the costs of those with earlier disease. Patients with stage IV disease at the start of treatment had costs similar to those with stage III, despite shorter survival. These findings may help plan healthcare budgets and support efforts to optimise treatment duration.

## 1. Introduction

Cutaneous melanoma is a worldwide health issue, with varying incidence rates. In 2024, the global age-standardised incidence rate was 3.1 per 100,000, with the highest rates observed in Australia and New Zealand (37.0 per 100,000), followed by Northern and Western Europe and North America [1]. In Serbia, the rate was 6.2 per 100,000 in men and 5.8 in women in 2022, with stable trends since 2008 and a slight decline in mortality from 2008 to 2022, suggesting potential improvements in early detection and treatment outcomes [2].

Most patients with melanoma in Europe are diagnosed with localised disease in stage I or II, but recurrence or progression to regional or distant sites worsens prognosis, requires more intensive treatment, and increases healthcare costs, especially in those with distant metastases [3]. Modern systemic therapies include immune checkpoint inhibitors (ICIs) and, for BRAF V600-mutated tumours, BRAF/MEK-targeted therapies. According to the current European Society for Medical Oncology guidelines, anti-PD-1 therapy is recommended for patients with resected high-risk stage II melanoma and for those with resected stage III or IV melanoma with no evidence of disease. For resected stage III BRAF V600-mutated melanoma, dabrafenib combined with trametinib is an additional standard treatment option. Neoadjuvant ICI treatment is recommended for selected resectable stage III patients with macroscopic nodal metastases. For unresectable stage III or IV melanoma, first-line treatment options include PD-1 inhibitors alone or in combination with CTLA-4 or LAG-3 inhibitors, as well as BRAF/MEK inhibitors for BRAF V600 mutations [4].

Before the introduction of ICIs and targeted therapies in standard clinical practice, the costs of melanoma care resulted mainly from surgery, hospital stays, and recurrences of the disease. With modern systemic therapies, survival among patients with high-risk resected or advanced melanoma has improved, but overall treatment costs have also increased [5,6]. In Serbia, the Republic Health Insurance Fund mainly finances healthcare through compulsory health insurance. Immunotherapy and targeted therapy for melanoma are included in List C of the national List of Medicines, which comprises high-cost medicines reimbursed only for specified indications and administered in inpatient healthcare institutions [7]. These medicines are started based on the opinion of three specialists from designated tertiary centres and the Fund’s expert commission, and continuation requires the regular assessment of treatment response. They are provided without co-payment, so out-of-pocket payments are limited mainly to services obtained at private institutions.

There is a need for detailed real-world data on resource use to support effective healthcare planning, which would provide an evidence base for forecasting future healthcare needs and guiding resource allocation. Cost estimates based on guidelines and decision models may not capture actual treatment patterns, recurrence rates, adverse events, or their associated costs in routine clinical practice [8]. To identify the main cost drivers, bottom-up cost-of-illness analyses of direct medical resource use are particularly useful because they capture actual costs and their variation across disease stages, treatments, and outcomes [8,9]. However, data regarding the relationship between costs and clinical outcomes remain scarce for melanoma treated with immunotherapy and targeted therapy, especially from healthcare systems in Central and Eastern Europe [10]. Therefore, we aimed to estimate the direct medical costs of immunotherapy and targeted therapy for cutaneous melanoma and relate them to clinical outcomes, using real-world data on individual patients from a tertiary referral hospital that is one of the national reference centres for melanoma treatment in the Serbian public healthcare system.

## 2. Materials and Methods

### 2.1. Study Design and Setting

We conducted a retrospective, single-centre, observational, bottom-up cost-of-illness study of patients with cutaneous melanoma treated with ICIs and BRAF/MEK-targeted therapy at the Clinic for Dermatovenerology, Military Medical Academy, Belgrade, Serbia (the study centre), a tertiary referral centre for melanoma care within the Serbian public healthcare system, and reported in accordance with the applicable items of CHEERS 2022 [11] (Supplementary Checklist S1) following the recommendations for cost-of-illness studies by Larg and Moss [12].

For the purposes of this study, the start of systemic therapy was defined as the date on which an ICI or BRAF/MEK-targeted regimen was first administered, whether in the neoadjuvant, adjuvant, or unresectable/metastatic setting. At the study centre, anti-PD-1 therapy was used for metastatic melanoma from 2017, adjuvant nivolumab and dabrafenib plus trametinib from late 2019, adjuvant pembrolizumab from 2021, nivolumab plus ipilimumab from 2022, and neoadjuvant pembrolizumab from 2024. Before a regimen was reimbursed for this indication, some patients received it through clinical trials or donation programmes.

The study design was approved by the Ethics Committee of the Military Medical Academy (Approval No. 43/2022, dated 11 October 2022), and all patients provided written informed consent. The study was conducted within the ReDiMEL project, funded by the Science Fund of the Republic of Serbia.

### 2.2. Study Population and Analytic Cohorts

All consecutive patients aged 18 years and older with histopathologically confirmed cutaneous melanoma who received immunotherapy and/or targeted therapy at the study centre during the cost observation window, from 1 January 2019 to 30 April 2026, were identified (*n* = 186), including patients whose systemic therapy had started before 2019. Three patients were excluded because their costs could not be reliably reconstructed due to insufficient information on prior or current therapy. This created a full analytic cohort of 183 patients, primarily for descriptive analysis of the entire study population. Within the full cohort, two nested cohorts were formed based on the start dates of follow-up and cost measurement. The primary cohort (*n* = 165) comprised patients diagnosed on or after 1 January 2019 and was used for analyses from diagnosis onward. Because inclusion required receipt of systemic therapy, this cohort represents patients who subsequently received systemic therapy rather than all patients diagnosed with melanoma. The secondary cohort (*n* = 179) was used for analyses from the start of systemic therapy and included all patients from the primary cohort plus 14 patients diagnosed before 2019 whose systemic therapy started on or after 1 January 2019. The remaining four patients had been diagnosed and had started systemic therapy before 2019. Because both relevant start dates for these patients fell before the cost observation window, they were excluded from the nested cohorts but remained in the full analytic cohort.

### 2.3. Cost Analysis

The cost observation window started on 1 January 2019, when itemised billing records became available, and ended on 30 April 2026, the date of the administrative data cut-off.

The analysis was conducted from a healthcare-sector perspective, which includes all direct medical costs regardless of payer, including out-of-pocket payments for medical services. To capture the complete cost of care, in addition to resources provided and billed by the study centre, which accounted for most care, we included services provided at other institutions or funded by other sources, such as open-label clinical trials, donation programmes, or out-of-pocket payments at private institutions, when they were documented in discharge letters and their costs could be reliably reconstructed.

Costs were estimated using a bottom-up, predominantly micro-costing approach [13], in which resources were identified and valued for each patient, summed to obtain the total cost per patient, and then averaged across patients. Melanoma-related healthcare resources were identified for each patient from hospital invoices, discharge letters, and the clinical database, and valued hierarchically using: (1) patient-specific invoice prices, (2) prices for the same resource elsewhere in the cohort, or (3) institutional tariffs. Medicines supplied through open-label clinical trials or donation programmes, typically before they were reimbursed for the given indication or disease stage, were valued at the price recorded for the same drug in other patients of the cohort during the same calendar period. Systemic therapy was micro-costed by multiplying the number of vials or tablets administered in each cycle by the unit price. Diagnostic procedures, laboratory tests, consultations, surgical procedures, molecular diagnostics, and other medications and consumables were recorded as individual services and valued at invoice prices or institutional tariffs. Inpatient stays, day-hospital visits, and administration of systemic therapy were valued using per-day or per-cycle institutional tariffs (gross costing). Personnel, overhead, and equipment costs were included in these prices and tariffs and were not itemised separately.

Radiotherapy costs were excluded because radiotherapy was provided both at the study centre and at external institutions, was not captured in the invoices for all patients, and could not be reliably reconstructed from the discharge letters. Non-medical and indirect costs were excluded.

For each patient, all direct medical cost items were calculated, classified into eight categories (immunotherapy and targeted therapy; radiological and nuclear diagnostics; inpatient and day-hospital care and other medications; surgery and histopathology; laboratory tests; consultations; molecular diagnostics, including BRAF testing and immunohistochemistry; and tumour markers and endocrine tests, including S-100 and thyroid function tests), and summed to obtain the total direct medical cost per patient. The contribution of each category to total costs was then analysed.

Costs were recorded in nominal Serbian dinars (RSD) at the prices applicable at the time each service was provided. Costs were not discounted because the analysis examined costs already incurred rather than projected future costs. RSD amounts were converted to euros (EUR) using the National Bank of Serbia’s official middle rate on 31 December 2025 (1 EUR = 117.2820 RSD). A single rate was used because the average annual EUR/RSD rate varied by less than 0.6% over 2019–2025. Inflation adjustments were not made because Serbia’s consumer price index excludes public-sector prices for systemic therapies and institutional tariffs, and no healthcare-specific index is available.

### 2.4. Outcomes and Covariates

Clinical outcomes included overall survival (OS), real-world progression-free survival (rwPFS), and time to next treatment or death (TNT-D). OS was defined as the time to death from any cause or to last contact, calculated from the date of histopathological diagnosis in the primary cohort and from the start of systemic therapy in the secondary cohort. rwPFS was defined as the time from the start of systemic therapy to either radiologically confirmed disease progression per RECIST v1.1 criteria or death from any cause, whichever occurred first [14]. TNT-D was defined as the time from the start of systemic therapy to the initiation of the next line of systemic therapy or death. Patients without the respective event were censored at the date of their last known contact. The duration of first-line therapy, number of cycles, and reasons for discontinuing treatment were obtained from the clinical database. Patients still receiving treatment at the data cut-off were censored. Systemic therapy at the end of life was defined as the end of the last line of systemic therapy within 90 days before death.

Stage at diagnosis and stage at the start of systemic therapy were classified according to the TNM system (8th edition) of the American Joint Committee on Cancer (AJCC) Cancer Staging Manual, based on histopathological and imaging findings [15]. First-line therapy intent was classified as neoadjuvant/perioperative (before planned resection), adjuvant (after complete resection with no evidence of disease), or metastatic (unresectable stage III or stage IV disease). BRAF mutation status was obtained from routine molecular diagnostics.

Baseline covariates included age at diagnosis, sex, stage at diagnosis, and BRAF mutation status.

Immune-related adverse events (irAEs) were recorded descriptively as present if at least one treatment-related immune-mediated event, regardless of grade, was documented in discharge letters or the clinical database.

For the phase-of-care analyses, follow-up from diagnosis was divided into three phases using a modified standard model. The initial phase comprised the first six months after diagnosis, the terminal phase the last six months before death, and the continuing phase comprised the period between them. If the initial and terminal phases overlapped, patient-months were assigned to the terminal phase [16,17,18]. For treatment-period analyses, follow-up was divided into the period before the start of systemic therapy, during first-line therapy (until the start of the next line or death), and after first-line therapy.

The primary cost outcome was the mean cumulative total direct medical cost per patient over the first 24 months from each predefined analysis anchor. The 24-month period captured early disease management and systemic treatment while providing sufficient follow-up for cost estimation. Additional estimates were calculated at 12, 36, 48, and 60 months, where follow-up data were available. Cost intensity was calculated as cost per patient-month for analyses by phase of care and treatment period.

### 2.5. Statistical Analysis

Categorical variables were presented as counts and percentages. Continuous variables were presented as mean ± standard deviation (SD) or median (interquartile range (IQR)).

OS, rwPFS, and TNT-D were estimated using the Kaplan–Meier method. Medians and 24-month estimates were reported with 95% confidence intervals (CIs). Differences between groups were assessed using the log-rank test. Median follow-up was estimated using the reverse Kaplan–Meier method, with censoring treated as the event. In a sensitivity analysis, OS from diagnosis was re-estimated in the full analytic cohort using the Kaplan–Meier method with delayed entry, where patients diagnosed before 1 January 2019 entered the risk set on that date, and all others on their diagnosis dates. The 24-month restricted mean survival time (RMST) was estimated as the area under the Kaplan–Meier curve, and the cost per month alive as the ratio of the IPCW-adjusted 24-month mean cost to the RMST.

Mean cumulative costs, overall and by cost category and subgroup, were estimated using the simple inverse-probability-of-censoring-weighted (IPCW) estimator of Bang and Tsiatis [19]. Only patients whose costs were fully observed up to the horizon contributed, each weighted by the inverse probability of remaining uncensored, which was estimated using the Kaplan–Meier method with censoring as the event. For subgroups, weights were re-estimated within each subgroup. Unadjusted and complete-case means were reported for comparison. Medians and interquartile ranges of 24-month costs were estimated from the IPCW-weighted distribution of fully observed costs. Estimates were not interpreted when more than 50% of patients in the cohort were censored before the horizon, based on the observed proportion of censored patients [20,21]. The Kaplan–Meier probability of remaining uncensored at each horizon, $\hat{K}$(*t*), was also reported.

Cost per patient-month was calculated as the total cost for each phase of care or treatment period divided by the total patient-months observed in that period. Cost per patient-month ratios were calculated relative to the period before the start of systemic therapy.

In the sensitivity analyses, 24-month costs were re-estimated using the partitioned Bang–Tsiatis estimator with 6-month intervals and separately with censoring weights re-estimated within calendar periods of the anchor date (2019–2021, 2022–2023, and 2024–2025). The simple IPCW estimate was used as the base case. The independence of censoring and costs was tested by simulation (one-sided Monte Carlo test, 400 replications, giving a smallest possible *p*-value of 0.0025), comparing the observed difference between the partitioned and simple estimates with differences obtained under simulated independent censoring.

In the primary cohort, associations of stage at diagnosis, BRAF status, age (per 10 years), and sex with 24-month costs and with OS from diagnosis were assessed using gamma generalised linear models with a log link and IPCW, and Cox proportional hazards models, respectively, following Haining et al. [22], where covariates for multivariable models were selected based on the literature and clinical relevance rather than univariable significance. Multivariable models included stage, BRAF status, and age, and patients with missing data were excluded. The proportional hazards assumption was tested using scaled Schoenfeld residuals, and when it was not met, hazard ratios were estimated post hoc separately for the first 12 months and thereafter. Because inclusion required receipt of systemic therapy, survival from diagnosis included the immortal time between diagnosis and the start of systemic therapy. Cox models were therefore fitted in the secondary cohort with delayed entry, in which patients entered the risk set at the start of systemic therapy.

CIs for cost estimates were obtained by patient-level bootstrap (1000 resamples and 10,000 for cost ratios), with censoring weights re-estimated in each resample. Except for the Monte Carlo test, all tests were two-sided, with *p* < 0.05 considered statistically significant.

Analyses were performed in Python 3.12 (NumPy 2.4.4, pandas 3.0.2, statsmodels 0.15.0, lifelines 0.30.3) and R 4.6.1 (survival 3.5.8, ccostr 0.1.1) [23]. GenAI (Claude by Anthropic, models Opus 5, Opus 5.5, and Fable 5) was used to assist with code development. The analysis code is available from the corresponding author on request.

## 3. Results

### 3.1. Study Cohorts, Patient Characteristics, Follow-Up, and Observed Costs

**Figure 1:**
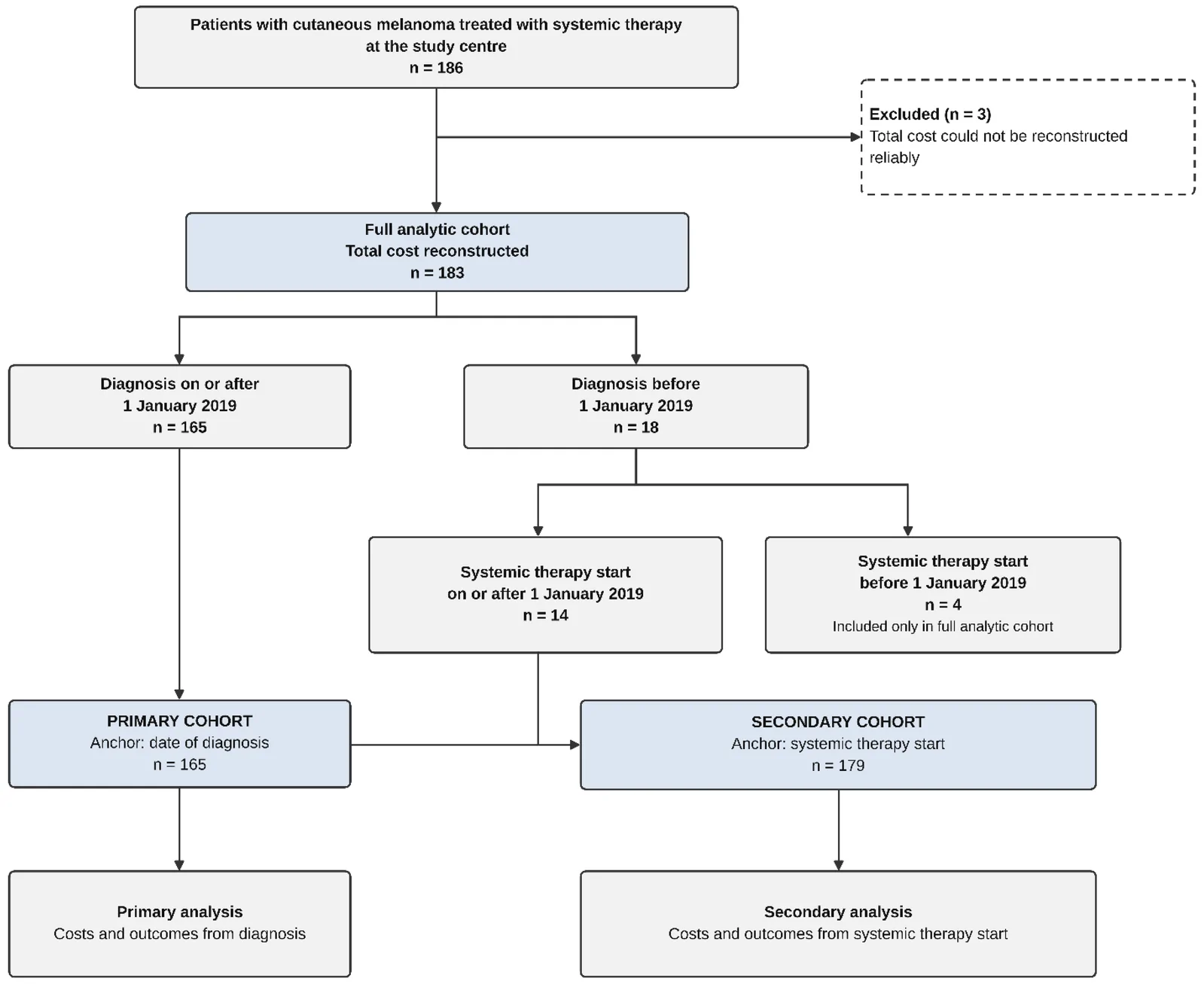
Flowchart of patient selection and definition of the two analytic cohorts.

Of the 186 consecutive melanoma patients receiving systemic therapy at the study centre, 183 had complete cost reconstructions and formed the full analytic cohort. Of these, 165 and 179 formed the primary and secondary cohorts, respectively. All patients in the primary cohort were also included in the secondary cohort, and the remaining four were included only in the full analytic cohort (Figure 1, Table 1).

Demographic, clinical, and treatment characteristics, as well as follow-up data of the study cohort, are presented in Table 1. In the primary cohort, the median age at diagnosis was 66 years, and almost 70% of patients were male. More than half of the patients had stage III disease at diagnosis, and 44.8% had a BRAF mutation. Pembrolizumab monotherapy was the most common first-line regimen (58.2%), followed by nivolumab (27.9%), and combination immunotherapy and BRAF/MEK inhibition accounted for 13.9%. Two-thirds of patients received only one line of systemic therapy. The median interval from diagnosis to the start of systemic therapy was 6.5 months (IQR 3.5–15.1). Overall, 73.3% of patients had irAEs.

Patients diagnosed before 2019 (n = 18) were younger, more often had stages I–II disease and BRAF mutations, and started systemic therapy much later after diagnosis than those in the primary cohort, reflecting survivor selection because they could enter the cohort only if they survived to the start of the cost observation (Table 1).

### 3.2. Distribution of Costs by Category, Phase of Care, and Treatment Period

In the primary cohort, the mean cumulative direct medical cost per patient over the first 24 months from diagnosis was EUR 66,466 (95% CI 58,465–75,334) (Table 2). Immunotherapy and targeted therapy accounted for EUR 62,970 (94.7%), and all other categories together for EUR 3496 (5.3%). These other categories represented only a small proportion of the total costs but were incurred by most patients. All patients had surgery and histopathology costs, and radiological and nuclear diagnostics and consultations in 92.7% of cases. Twenty patients (12.1%) had no immunotherapy or targeted therapy costs within this horizon because their systemic therapy started more than 24 months after diagnosis.

The distribution of total direct medical costs in the full analytic cohort (*n* = 183) over the entire observed follow-up by unit-cost source is presented in Supplementary Table S1. Overall, 84.0% of costs were valued using prices observed in the payment system, either patient-specific invoice prices (28.5%) or prices observed elsewhere in the cohort (55.5%), while the remaining 16.0% were valued using institutional tariffs. By funding source, 94.2% of costs were covered by the national health insurance fund, 3.1% by donation programmes (7 patients), 2.4% by clinical trial sponsors (2 patients), and 0.3% by out-of-pocket payments (6 patients). These out-of-pocket payments were mostly for systemic therapy received by two patients at private institutions, and for excision of the primary tumour, imaging, or consultations for the other four patients.

**Figure 2:**
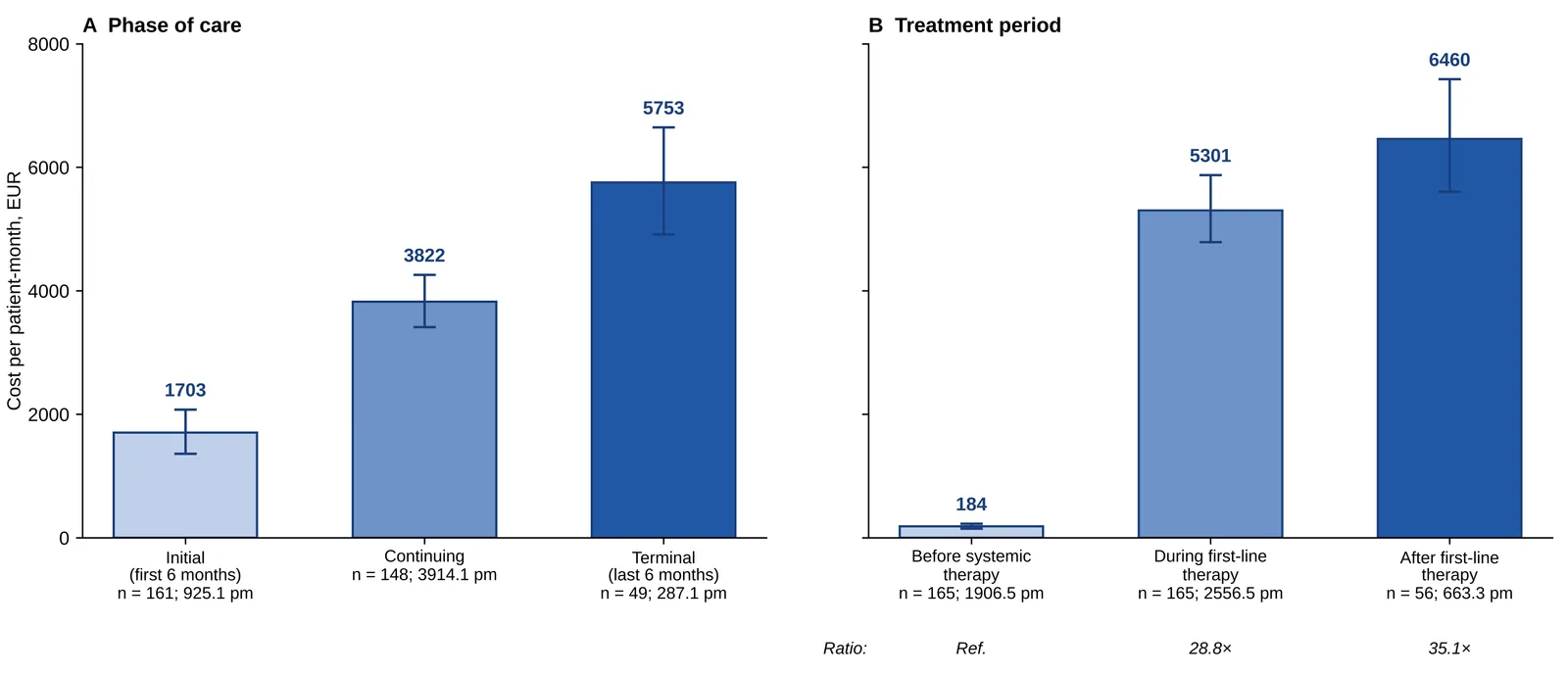
Cost per patient-month by (**A**) phase of care and (**B**) treatment period in the primary cohort (*n* = 165) from diagnosis to death or end of follow-up. Note. Bars show cost per patient-month, and error bars show 95% bootstrap confidence intervals (CIs); the number of patients and total observed patient-months are shown below each bar. Phases of care were defined as initial (the first 6 months after diagnosis), terminal (the last 6 months before death), and continuing (the period in between); overlaps between the initial and terminal phases were assigned to the terminal phase. Treatment periods were defined as before the start of systemic therapy, during first-line therapy (until the start of the next line or death), and after first-line therapy. Ratios in 2B are relative to the period before the start of systemic therapy. Detailed values are given in Supplementary Table S2. Abbreviations: CI, confidence interval; EUR, euro; pm, patient-months; Ref., reference period.

Within the primary cohort, follow-up was divided by phase of care and by treatment period (Figure 2 and Supplementary Table S2). Cost per patient-month increased across phases of care, from EUR 1703 in the initial phase to EUR 3822 in the continuing phase and EUR 5753 in the terminal phase. Because systemic therapy was initiated at a median of 6.5 months after diagnosis (Table 1), much of the initial phase occurred before treatment began. By treatment period, cost per patient-month increased from EUR 184 before systemic therapy to EUR 5301 during first-line therapy and EUR 6460 after first-line therapy, 28.8 and 35.1 times higher, respectively.

### 3.3. Cumulative Costs by Analysis Anchor and Time Horizon

Over a period of 24 months, the IPCW-adjusted mean cumulative cost per patient was EUR 66,466 (95% CI 58,465–75,334) from diagnosis in the primary cohort and EUR 107,229 (95% CI 97,405–118,201) from the start of systemic therapy in the secondary cohort (Table 3), showing that the treatment-anchored estimate was 61% higher than the diagnosis-anchored estimate. The corresponding IPCW-weighted medians were EUR 68,460 (IQR 16,030–103,140) from diagnosis and EUR 107,330 (IQR 61,530–143,760) from treatment start, close to the means, indicating that the 24-month costs were not markedly right-skewed. The difference between the two anchors was mainly attributable to the different periods covered by the two horizons. Because systemic therapy started a median of 6.5 months after diagnosis (as shown in Table 1), the diagnosis-anchored horizon included a pre-treatment period with low costs (EUR 184 per patient-month; Supplementary Table S2) and fewer months of systemic therapy, and 20 patients (12.1%) received no systemic therapy within 24 months of diagnosis. In contrast, the treatment-anchored horizon covered 24 months from the start of systemic therapy, when costs reached EUR 5301 per patient-month during first-line therapy. Both analyses used the same cost categories and estimator.

Compared with the unadjusted mean, the IPCW-adjusted 24-month cost was 12.6% higher at the treatment anchor (EUR 107,229 vs. 95,266) and 6.2% lower at the diagnosis anchor (EUR 66,466 vs. 70,879). The IPCW-adjusted mean from diagnosis was lower than the unadjusted mean because the unadjusted mean included the partial costs of censored patients, most of whom were diagnosed in 2024–2025 and had higher costs, whereas the simple estimator used only fully observed patients, mostly diagnosed earlier (Supplementary Table S3B). At 24 months, 23.0% of patients in the primary cohort and 41.3% in the secondary cohort were censored before the horizon, with probabilities of remaining uncensored, $\hat{K}$(24), of 0.735 and 0.460, respectively.

In the sensitivity analyses, the 24-month base-case estimate of mean costs increased by up to 17.2% from diagnosis (from EUR 66,466 to 77,901) and by up to 5.1% from the start of systemic therapy (from EUR 107,229 to 112,734) (Supplementary Table S3). The simulation test indicated that censoring depended on costs from diagnosis (0 of 400 simulations; *p* < 0.003) but not from the start of systemic therapy (*p* = 0.22), and suggested that the base case may underestimate costs from diagnosis.

### 3.4. Associations of Patient Characteristics with Costs and Survival

Stage at diagnosis was the only baseline characteristic associated with 24-month cost (Table 4). After adjustment for BRAF status and age, mean costs were 2.19-fold higher in stage III and 1.91-fold higher in stage IV than in stages I–II. Similar costs were observed between patients in stages III and IV at diagnosis, but stage IV was associated with higher mortality (hazard ratio (HR) 6.40, 95% CI 2.75–14.89), while mortality in stage III was comparable to that in stages I–II (HR 1.04, 95% CI 0.46–2.32). With delayed entry at the start of systemic therapy, the HR for stage IV decreased to 2.93 (95% CI 1.21–7.12), and mortality in stage III remained comparable to that in stages I–II (HR 0.61, 95% CI 0.27–1.39). BRAF status, age, and sex were not associated with cost or mortality.

### 3.5. Clinical Outcomes and Stage-Related Cost Patterns

From the start of systemic therapy, 24-month OS was 69.2% compared with 46.2% for rwPFS and 47.2% for TNT-D, suggesting that many patients survived long after progression or the start of subsequent therapy (Table 5).

Outcomes differed by first-line therapy intent (all *p* < 0.001; Table 5). At 24 months, OS was 91.5% in the adjuvant group and 51.9% in the metastatic group; rwPFS was 62.4% and 33.0%, and TNT-D was 60.4% and 36.0%, respectively. In the neoadjuvant/perioperative group (*n* = 14), 24-month estimates could not be obtained because no patients remained at risk at 24 months. The median duration of first-line therapy was 11.1 months (IQR 9.1–12.0) with adjuvant intent and 6.8 months (IQR 2.5–32.4) with metastatic intent, with a median of 9.0 cycles (IQR 5.0–14.0) (Supplementary Table S4). Of the 68 adjuvant patients who completed first-line therapy, 43 (63.2%) completed it as planned, 14 (20.6%) stopped because of progression, and 7 (10.3%) because of toxicity. Of the 55 patients who died, 52 (94.5%) received systemic therapy in the last 90 days of life.

**Figure 3:**
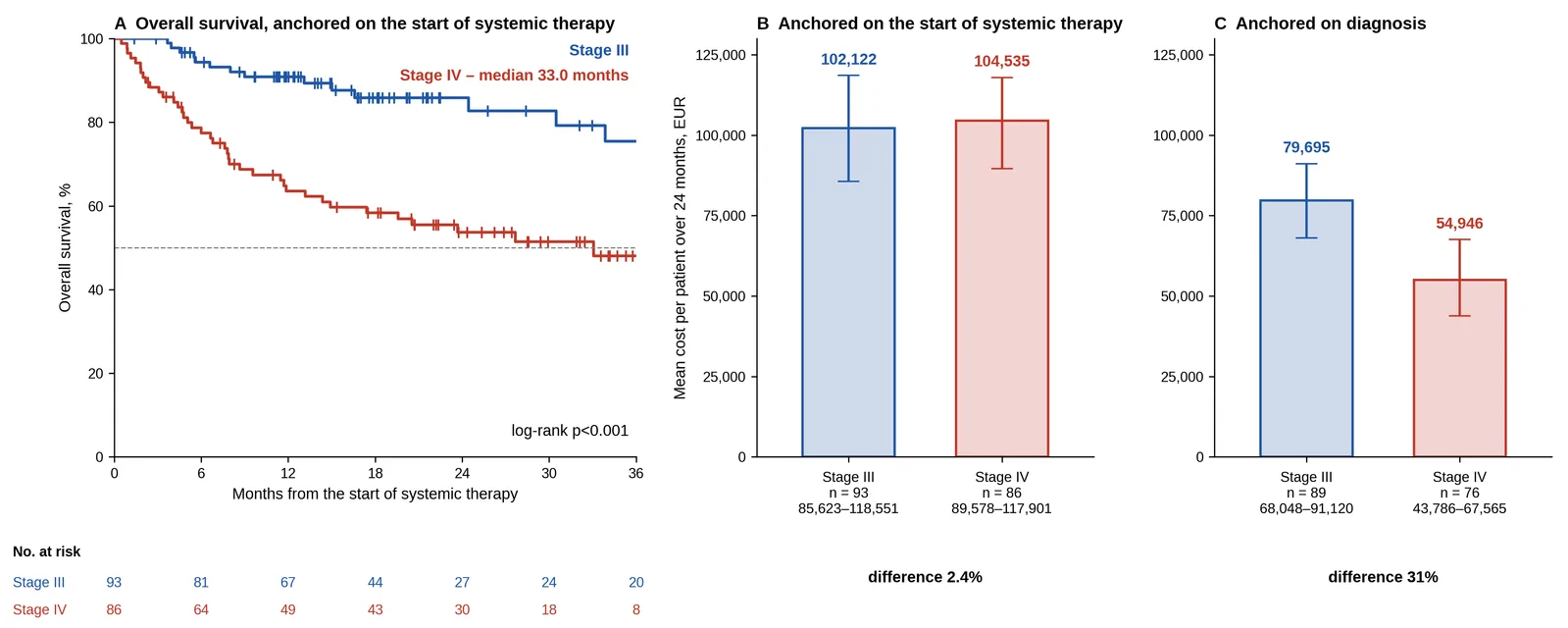
Overall survival and 24-month cumulative costs by stage at the start of systemic therapy. (**A**) Kaplan–Meier overall survival curves for stage III and stage IV disease, anchored at the start of systemic therapy; tick marks indicate censored observations; (**B**) IPCW-adjusted mean cumulative cost over 24 months from the systemic therapy start; (**C**) IPCW-adjusted mean cumulative cost over 24 months from diagnosis. Note. A and B refer to the secondary cohort (*n* = 179) and C to the primary cohort (*n* = 165). Bars show IPCW-adjusted 24-month mean cumulative costs with 95% confidence intervals. Because censoring weights were estimated within each stage group, stage-specific estimates may not match the overall estimate. Abbreviations: EUR, euro; IPCW, inverse probability of censoring weighting.

By stage at the start of systemic therapy, 24-month mean costs from the start of systemic therapy were similar in stage III and stage IV (EUR 102,122 vs. 104,535; difference 2.4%), despite markedly shorter OS in stage IV (median 33.0 months; log-rank *p* < 0.001) (Figure 3A,B). Over the first 24 months of systemic therapy, patients with stage IV disease lived 5.3 months less on average than those with stage III (RMST 16.6 vs. 21.9 months; *p* < 0.001), so their cost per month alive was 35% higher (EUR 6306 vs. 4664). From diagnosis, the 24-month mean costs were 31% lower in stage IV than in stage III (EUR 54,946 vs. 79,695) (Figure 3C).

## 4. Discussion

This study analysed direct medical costs from a healthcare-sector perspective for patients with cutaneous melanoma treated with ICIs or BRAF/MEK-targeted therapy in a public healthcare system. Mean 24-month direct medical costs were EUR 66,466 from diagnosis and EUR 107,229 from the start of systemic therapy, and immunotherapy and targeted therapy were the main cost drivers, accounting for 94.7% of 24-month costs from diagnosis. Costs increased considerably when systemic therapy began and remained high after first-line treatment. Only stage at diagnosis was associated with costs. Mean costs from the initiation of systemic therapy were similar between stages III and IV, although survival was substantially shorter in stage IV.

In the period before ICIs and BRAF/MEK-targeted therapy, systemic therapy accounted for only 18% of melanoma costs, even in the treatment of distant metastases, while most costs came from hospital visits, admissions, and surgery [3]. In our cohort, it accounted for 94.7% of costs over 24 months from diagnosis, similar to results reported by Buja et al., whose estimates were based on an Italian guideline-based model, which reported 92.0% for stage IV and 86.5% for stage III with in-transit metastases [24].

Other studies also found that systemic therapy accounted for the largest share of costs. Franken et al. reported 84% of hospital costs for Dutch patients with metastatic melanoma [25], while in Canada, Krebs et al. reported up to 73.6% for patients with advanced melanoma [26] and Lu et al. 57% for those receiving second-line ipilimumab [27]. Our higher share most likely reflects differences in the patient population and included costs, as all our patients received systemic therapy, compared with 83% in the study by Franken et al. and about two-thirds in that by Krebs et al. Both Canadian studies included non-cancer care, and Lu et al. also included long-term and home care in costs. Among treated patients, costs were similar, EUR 118,905 per patient over a mean of 21.2 months in the study by Franken et al. vs. EUR 104,535 over 24 months from treatment start among our patients with stage IV disease at initiation.

The starting point for cost measurement had an impact on both the magnitude and the stability of our estimates. Mean 24-month costs were 61% higher when measured from the start of systemic therapy rather than from diagnosis (in largely overlapping cohorts), because the period after diagnosis often included months of surgery, staging, or surveillance before the more expensive systemic therapy began. Alternative censoring adjustments increased the estimates by up to 5.1% from the start of systemic therapy, but by up to 17.2% from diagnosis. The simulation test also indicated that censoring depended on costs from diagnosis, likely because recently diagnosed patients, who had the shortest follow-up, started systemic therapy sooner after diagnosis and therefore had higher early costs. Mean 24-month costs from diagnosis also increased across calendar periods, from EUR 48,422 in 2019–2021 to EUR 89,507 in 2024–2025 (Supplementary Table S3B), as adjuvant and combination regimens became available later in the study period. This also explains the low use of combination immunotherapy as first-line therapy. The simple weighted estimator becomes unstable as censoring increases, particularly above 50%, while the partitioned estimator, which also uses costs observed before censoring, is more stable [20,21]. For this reason, we did not interpret estimates at horizons where more than half of the patients had been censored. However, when censoring depends on calendar period, neither estimator is unbiased. We therefore used the simple estimator as a conservative base case, consistent with the IPCW weights in the regression and subgroup analyses, and report the partitioned estimate alongside it. The 24-month estimate from the start of systemic therapy remained stable, despite only a 0.46 probability of remaining uncensored at that point. It changed little with alternative censoring adjustments, and censoring did not appear to be associated with costs (*p* = 0.22). Since the base case gave the lowest of all estimates, the true 24-month costs are probably not lower than reported and may be higher.

Cost intensity in our cohort rose as soon as systemic therapy began, reaching EUR 5301 per patient-month during first-line therapy and EUR 6460 after it. These values were at or below the lower end of the monthly costs of continuous treatment with modern regimens estimated in Italy (EUR 6390–9453) [24], as our estimates were averaged over entire treatment periods, including months without active treatment. Across phases of care, costs in our cohort increased steadily and were highest in the terminal phase. Earlier studies, conducted before modern systemic therapies, found a U-shaped pattern, with the highest melanoma costs in the initial and terminal phases [17,28], low in the continuing phase, and rising sharply only in the last year of life [29]. The difference in the initial phase likely reflects the starting point, where Atkins et al. [28] measured costs from the diagnosis of metastatic disease, when treatment usually begins, whereas our initial phase started at first diagnosis, with systemic therapy beginning a median of 6.5 months later. The high costs after first-line therapy and at the end of life are consistent with continued systemic treatment late in the disease. In a German registry study, it was reported that of the 1067 patients with metastatic melanoma who died between 2018 and 2022, 73.6% received systemic therapy in the last 90 days of life, 45.2% in the last 30 days, and 39.1% had received at least three lines of therapy [30].

Stage at diagnosis was the only baseline characteristic associated with 24-month costs, which were about twice as high in stages III and IV as in stages I–II. Because the log-link gamma model estimates mean costs directly, these ratios compare mean costs without the retransformation needed after regression on log-transformed costs, which can be biased when cost variability differs between groups [31]. Although stage IV at diagnosis was associated with markedly higher mortality, its costs were not higher than those in stage III. Part of this mortality difference reflected immortal time, because patients diagnosed with stages I–II disease entered the cohort only after recurrence, a median of 27.2 months after diagnosis. With delayed entry at the start of systemic therapy, stage IV remained associated with higher mortality, but the HR decreased from 6.40 to 2.93. A similar pattern was observed by stage at the start of systemic therapy. From that point onward, 24-month costs were similar for stages III and IV, despite substantially shorter survival in stage IV (median OS 33.0 months). Similar total costs therefore reflected a higher intensity of resource use in stage IV, concentrated in a shorter survival time. Measured from diagnosis, costs in stage IV were even 31% lower, likely because many of these patients had been diagnosed at an earlier stage and started systemic therapy later, or died within the horizon. Other population-based studies found that costs rise substantially with stage, from AUD 2794 for *in situ* melanoma to AUD 70,070 for distant metastases in Australia [9], and from CAD 67,108 in stage III to CAD 117,450 in stage IV in Canada [5]. However, only 34–41% of patients in these studies received immunotherapy or targeted therapy, and treated patients had costs approximately AUD 60,000 per year higher than untreated patients [9], whereas all our patients received systemic therapy. The similar costs in stages III and IV in our cohort may also reflect adjuvant therapy in resected stage III disease, which shifts treatment costs to earlier stages [32].

Survival outcomes aligned with the benefits of modern systemic therapy. In our metastatic-intent group, median OS was 27.7 months, median rwPFS 8.4 months, and 2-year OS 51.9%, compared with 12.1 months, 2.7 months, and 34% in a Norwegian phase IV trial of ipilimumab, in which 44% of patients received PD-1 inhibitors after progression [33]. Similarly, after the adoption of immunotherapy and targeted therapy in Ontario, 3-year OS improved from 65.0% to 73.6% in stage III and from 16.1% to 32.1% in stage IV [5].

A recent review by Ngo et al. found that evidence of direct cancer costs in 39 countries was concentrated in high-income settings, with fully bottom-up estimates available for only nine countries. Also, they concluded that cancer expenditure grew faster than gross domestic product and that differences between countries largely reflected national income and price levels [10]. Before modern systemic therapies, treatment costs were markedly lower in Central and Eastern European countries than in Western Europe [34], whereas costs for our treated patients with stage IV disease were close to those in the Netherlands [25]. Moreover, the study by Franken et al. used list prices for drugs. Actual costs in the Netherlands were probably lower because hospitals negotiated drug prices, and nivolumab and pembrolizumab were among the 30 drugs covered by confidential national agreements, which reduced the overall costs by 36% in 2018 [25]. In Serbia, the actual costs are probably lower as well, since the Republic Health Insurance Fund negotiates drug prices through confidential agreements. Nevertheless, our findings suggest that unlike earlier melanoma care, modern systemic therapies cost about the same in lower- and higher-income countries and may therefore absorb a larger share of healthcare budgets in countries such as Serbia. As drug prices differ even among high-income countries [35], country-specific economic evaluations are needed.

Treatment in our cohort reflected changes in the Serbian national guideline, which aligns with international recommendations. The 2019 guideline, in effect during most of the study period, recommended 12 months of adjuvant anti-PD-1 therapy for resected stage IIIB, IIIC or IV disease, or dabrafenib plus trametinib for BRAF-mutated stage III disease. However, it noted that adjuvant therapy was not yet available in Serbia, and classified neoadjuvant therapy as investigational [36]. The 2026 updated national guideline extended adjuvant anti-PD-1 therapy to resected stage IIB and IIC disease and added neoadjuvant nivolumab plus ipilimumab and perioperative pembrolizumab for stage III disease, as well as nivolumab plus relatlimab for metastatic disease [37]. Because no patient in our cohort started systemic therapy at stage II, broader use of adjuvant therapy for stage IIB and IIC disease may shift costs to earlier stages.

Because systemic therapy accounts for most costs, shorter treatment could lead to substantial savings. A model-based study estimated that stopping ipilimumab early in patients who responded to treatment would save USD 19,891 per patient with advanced melanoma [38]. The ongoing randomised Grand SLAM trial is testing whether 6 months of adjuvant anti-PD-1 therapy is non-inferior to 12 months, and will also assess cost savings [39]. This question is relevant to our setting, as 42% of patients in our secondary cohort started systemic therapy with adjuvant intent. In our cohort, one-quarter of patients treated with metastatic intent remained on first-line therapy for more than 32 months, and almost all patients who died received systemic therapy in the last 90 days of life, which contributed to the high terminal-phase costs. Shorter treatment could also reduce the burden on patients, since those with stage IV disease spend more than one day per week in contact with the healthcare system during the first year of treatment, as reported by Bateni et al. [5]. In a Japanese real-world analysis, circulating tumour DNA (ctDNA) monitoring has been proposed as a way to identify patients in whom ICI could be safely stopped, avoiding prolonged ICI treatment and its disproportionate share of drug costs [40]. Analyses of ctDNA in our cohort are still underway, and will be linked to the cost data in the future.

One of the approaches that could substantially reduce treatment costs is neoadjuvant therapy for macroscopic nodal disease, in which patients receive only two cycles of nivolumab plus ipilimumab followed by surgery, and adjuvant therapy is omitted in those with a major pathological response [41]. This approach reduced the risk of progression, recurrence, or death compared with surgery followed by adjuvant therapy and may also reduce costs. In our study, only 14 patients were treated with this approach, mostly perioperatively, so no conclusions regarding costs and survival can be drawn at this point, and future studies are warranted.

These findings have several implications for healthcare decision-makers. Because systemic therapy accounted for nearly all costs, and 24-month costs from the start of treatment were similar for stages III and IV, budget planning should be based on the expected number of patients starting systemic therapy, at about EUR 107,000 per patient over two years. The extension of adjuvant therapy to stages IIB and IIC disease recommended in the current national guideline [37] will increase the number of treated patients and should be anticipated in budget planning. Drug prices and treatment duration also drive costs, supporting continued price negotiations, evaluation of shorter adjuvant therapy and response-based stopping rules, and broader use of short neoadjuvant regimens. Costs remained high after first-line therapy and in the terminal phase, as almost all patients who died received systemic therapy in the last 90 days of life, which should also be taken into account.

### Strengths and Limitations

The main strengths of this study are its design, which allowed us to follow patients from diagnosis through subsequent treatment periods, the valuation of the majority of costs using prices observed in the payment system, and censoring-adjusted estimates based on two clinically relevant anchors. Micro-costing is most transparent when resource quantities and unit costs are reported separately [13,42]. In our study, personnel, equipment, and overhead costs for non-drug services were captured only through institutional tariffs, so the approach was predominantly micro-costed, as in most published micro-costing studies [13]. However, these services accounted for only 5.3% of the 24-month costs, so this likely had little effect on the total cost estimates. Services received outside the study centre were captured only when documented in discharge letters and may therefore be incomplete, particularly out-of-pocket payments for services obtained at private institutions, which are likely to be underreported.

In our study, radiotherapy costs could not be reliably reconstructed and were excluded. Radiotherapy was received by 20.2% of patients, more often by those with stage IV than stage III disease at the start of systemic therapy (32.6% vs. 9.3%), so its exclusion underestimates stage IV and terminal-phase costs more than stage III costs.

The 24-month horizon does not reflect the long-term costs of chronic and late-onset immune-related adverse events, which may include lifelong hormone replacement therapy, prolonged specialist care or hospitalisations, immunosuppression, and rehabilitation [43].

Other limitations include the single-centre retrospective design and the use of nominal prices. However, as one of the national reference centres for melanoma treatment, the study centre treats patients from across Serbia, so our cohort may be broadly representative of patients receiving systemic therapy for melanoma in Serbia, although patient selection may differ between centres. In Serbia, systemic therapy for melanoma is prescribed according to the national and international guidelines [4,37] and the reimbursement criteria of the national health insurance fund, which apply to all centres, so differences between centres are more likely to reflect patient selection and referral than treatment regimens. Because inclusion required receipt of systemic therapy, diagnosis-anchored costs and survival apply only to patients who later received systemic therapy. Finally, this cost-of-illness study does not show whether these therapies are cost-effective in Serbia, which would require comparative economic evaluations.

## 5. Conclusions

This real-world analysis shows that modern systemic melanoma treatments are associated with substantial direct medical costs, with a mean of EUR 66,466 per patient over 24 months from diagnosis (EUR 75,388 with the partitioned estimator), 94.7% of which was attributable to immunotherapy and targeted therapy. Costs were about twice as high in patients with stage III or IV disease at diagnosis as in those with stages I–II disease. Direct medical costs from the start of systemic therapy were similar in patients in stages III and IV, but costs per month alive were 35% higher in stage IV because of poorer survival. Cost estimates depend strongly on the starting point of measurement, which should therefore be reported explicitly in cost-of-illness studies. Strategies to optimise treatment duration, including biomarker-guided approaches, require further evaluation. Budget planning should be based on the expected number of patients starting systemic therapy and should account for the extension of adjuvant therapy to earlier stages.

## Figures and Tables

**Table 1:** Demographic, clinical and treatment characteristics and follow-up of the study cohort.

Characteristic	Primary Cohort (*n* = 165)	Patients Diagnosed Before 2019 (*n* = 18)	Full Analytic Cohort (*n* = 183)
Patients, *n*	165	18	183
Included in the secondary cohort (anchor at systemic therapy start), *n* (%)	165 (100.0)	14 (77.8)	179 (97.8)
**Demographics**			
Age at diagnosis, years, median (IQR)	66 (51–73)	48 (38–60)	63 (50–72)
Male sex, *n* (%)	115 (69.7)	8 (44.4)	123 (67.2)
**Stage at diagnosis,** ***n*** **(%)**			
I	6 (3.6)	5 (27.8)	11 (6.0)
II	33 (20.0)	9 (50.0)	42 (23.0)
III	93 (56.4)	3 (16.7)	96 (52.5)
IV	29 (17.6)	1 (5.6)	30 (16.4)
Unknown	4 (2.4)	0 (0.0)	4 (2.2)
**BRAF status,** ***n*** **(%)**			
Mutated	74 (44.8)	15 (83.3)	89 (48.6)
Wild-type	88 (53.3)	3 (16.7)	91 (49.7)
Not tested	3 (1.8)	0 (0.0)	3 (1.6)
**Treatment**			
**First** -**line systemic therapy,** ***n*** **(%)**			
Pembrolizumab	96 (58.2)	7 (38.9)	103 (56.3)
Nivolumab	46 (27.9)	4 (22.2)	50 (27.3)
Dabrafenib + trametinib	11 (6.7)	4 (22.2)	15 (8.2)
Nivolumab + ipilimumab	10 (6.1)	0 (0.0)	10 (5.5)
Pembrolizumab + ipilimumab	2 (1.2)	1 (5.6)	3 (1.6)
Vemurafenib + cobimetinib	0 (0.0)	1 (5.6)	1 (0.5)
Other/clinical trial	0 (0.0)	1 (5.6)	1 (0.5)
**Stage at the start of systemic therapy,** ***n*** **(%)**			
III	89 (53.9)	5 (27.8)	94 (51.4)
IV	76 (46.1)	13 (72.2)	89 (48.6)
**First-line therapy intent,** ***n*** **(%)**			
Neoadjuvant/perioperative	14 (8.5)	0 (0.0)	14 (7.7)
Adjuvant	72 (43.6)	4 (22.2)	76 (41.5)
Metastatic	79 (47.9)	14 (77.8)	93 (50.8)
**Systemic therapy lines received,** ***n*** **(%)**			
1	109 (66.1)	6 (33.3)	115 (62.8)
2	43 (26.1)	6 (33.3)	49 (26.8)
3	12 (7.3)	4 (22.2)	16 (8.7)
≥4	1 (0.6)	2 (11.1)	3 (1.6)
Immune-related adverse event, *n* (%)	121 (73.3)	17 (94.4)	138 (75.4)
Time from diagnosis to systemic therapy start, months, median (IQR)	6.5 (3.5–15.1)	60.1 (33.7–70.8)	8.3 (3.7–17.7)
**Outcomes and follow-up**			
Died, *n* (%)	49 (29.7)	8 (44.4)	57 (31.1)
Censored, *n* (%)	116 (70.3)	10 (55.6)	126 (68.9)
Follow-up from date of diagnosis, months, median (IQR)	34.8 (23.0–52.6)	115.5 (91.6–130.4)	38.6 (24.5–59.8)
Cost observation period, months, median (IQR) ^a^	26.7 (18.6–44.2)	85.2 (63.3–87.6)	31.1 (19.7–50.1)
Total observed cost per patient, all categories, EUR, mean (SD) ^b^	110,223 (71,626)	207,933 (146,619)	119,834 (86,500)
Total observed cost per patient, all categories, EUR, median (IQR) ^b^	102,184 (57,676–143,097)	189,825 (73,557–331,997)	102,377 (57,778–154,302)
Observed cost per patient per month of observation, EUR, median (IQR) ^a^	3964 (2268–5339)	2677 (888–3779)	3832 (2031–5226)
**Year of diagnosis,** ***n*** **(%)**			
≤2018	0 (0.0)	18 (100.0)	18 (9.8)
2019–2021	38 (23.0)	0 (0.0)	38 (20.8)
2022–2023	74 (44.8)	0 (0.0)	74 (40.4)
2024–2025	53 (32.1)	0 (0.0)	53 (29.0)

Note. No statistical comparisons were performed, as the full analytic cohort includes both other columns. ^a^ Measured from diagnosis or from 1 January 2019 for patients diagnosed before that date, to the end of follow-up. ^b^ Observed costs over each patient’s entire follow-up, unadjusted for censoring. Abbreviations: BRAF, B-Raf proto-oncogene serine/threonine kinase; EUR, euro; IQR, interquartile range; SD, standard deviation.

**Table 2:** Mean cumulative cost per patient by cost category over the first 24 months from diagnosis in the primary cohort (*n* = 165).

Cost Category	Mean Cost per Patient, EUR (95% CI)	% of Total	Patients with Cost in Category Within 24 Months, *n* (%)
Immunotherapy and targeted therapy	62,970 (55,252–71,533)	94.7	145 (87.9)
Radiological and nuclear diagnostics	1166 (1005–1344)	1.8	153 (92.7)
Inpatient and day-hospital care and other medications	968 (810–1153)	1.5	149 (90.3)
Surgery and histopathology	715 (586–856)	1.1	165 (100.0)
Laboratory tests	307 (232–399)	0.5	121 (73.3)
Consultations	148 (121–176)	0.2	153 (92.7)
Molecular diagnostics	118 (106–130)	0.2	143 (86.7)
Tumour markers and endocrine tests	74 (57–92)	0.1	92 (55.8)
Total	66,466 (58,465–75,334)	100.0	165 (100.0)

Note. Means were estimated using the IPCW estimator over 24 months from diagnosis. The category means add up to the total. Abbreviations: CI, confidence interval; EUR, euro; IPCW, inverse probability of censoring weighting.

**Table 3:** Mean cumulative cost per patient by analysis anchor and time horizon, before and after censoring adjustment.

Analysis Anchor	Time Horizon, Months	Censored Before Horizon, *n* (%)	Probability of Remaining Uncensored, $\hat{K}$(*t*)	Unadjusted Mean Cost, EUR	Complete-Case Mean Cost, EUR	IPCW-Adjusted Mean Cost, EUR (95% CI)
From diagnosis (primary cohort, *n* = 165)	12	8 (4.8)	0.949	34,511	33,943	33,944 (28,836–38,656)
	24	38 (23.0)	0.735	70,879	65,671	66,466 (58,465–75,334)
	36	71 (43.0)	0.470	89,728	83,813	88,782 (75,306–102,580)
	48	87 (52.7)	0.323	101,467	93,178	106,481 (87,334–129,924)
	60	107 (64.8)	0.113	107,647	80,079	99,110 (79,343–121,475)
From the systemic therapy start (secondary cohort, *n* = 179)	12	25 (14.0)	0.836	73,255	75,900	77,155 (72,455–82,076)
	24	74 (41.3)	0.460	95,266	95,187	107,229 (97,405–118,201)
	36	98 (54.7)	0.255	106,998	102,802	136,722 (116,406–157,850)

Note. The unadjusted mean was calculated for all patients from the anchor date until death, censoring, or the time horizon, and the complete-case mean was calculated only for patients who died before the time horizon or were followed up to it. $\hat{K}$(*t*) is the Kaplan–Meier estimate of the probability of remaining uncensored up to time *t*, with censoring treated as the event. Shaded rows were not used in the primary interpretation because more than 50% of patients in the cohort were censored before the time horizon. The observed proportion of censored patients is lower than 1 − $\hat{K}$(*t*), because patients who died before the horizon could not be censored. The decrease in the IPCW-adjusted mean from 48 to 60 months from diagnosis reflects the instability of the simple estimator under heavy censoring. Abbreviations: CI, confidence interval; EUR, euro; IPCW, inverse probability of censoring weighting.

**Table 4:** Factors associated with 24-month cost and overall survival in the primary cohort (*n* = 165), and for delayed-entry models, the secondary cohort (*n* = 179).

Patient Characteristic	Cost Ratio for 24-Month Cost (95% CI)	*p*-Value	Mortality HR, from Diagnosis (95% CI)	*p*-Value	Mortality HR, Delayed Entry (95% CI)	*p*-Value
**Univariable analysis**						
Stage at diagnosis III vs. stages I–II	2.11 (1.47–3.46)	<0.001	1.14 (0.54–2.43)	0.73	0.70 (0.32–1.53)	0.38
Stage at diagnosis IV vs. stages I–II	1.94 (1.22–3.31)	0.005	6.15 (2.73–13.86)	<0.001	2.98 (1.27–7.01)	0.012
BRAF-mutated vs. wild-type	1.11 (0.87–1.43)	0.38	1.03 (0.58–1.84)	0.91	0.91 (0.51–1.60)	0.74
Age, per 10-year increase	0.96 (0.89–1.05)	0.36	1.12 (0.91–1.38)	0.28	1.15 (0.96–1.39)	0.14
Male vs. female	1.02 (0.79–1.38)	0.89	1.03 (0.55–1.89)	0.94	1.00 (0.56–1.77)	0.99
**Multivariable analysis**						
Stage at diagnosis III vs. stages I–II	2.19 (1.54–3.66)	<0.001	1.04 (0.46–2.32)	0.93	0.61 (0.27–1.39)	0.24
Stage at diagnosis IV vs. stages I–II	1.91 (1.20–3.37)	0.008	6.40 (2.75–14.89)	<0.001	2.93 (1.21–7.12)	0.018
BRAF-mutated vs. wild-type	1.12 (0.83–1.56)	0.44	1.14 (0.63–2.09)	0.66	1.02 (0.56–1.85)	0.96
Age, per 10-year increase	0.96 (0.86–1.06)	0.39	1.18 (0.94–1.48)	0.14	1.18 (0.97–1.45)	0.10

Note. Cost ratios were estimated using gamma generalised linear models with a log link and IPCW, and hazard ratios using Cox proportional hazards models. Multivariable models included 158 patients with complete data on stage and BRAF status, and 44 deaths were included in the survival model. In the multivariable survival model, the proportional hazards assumption was not met for stage IV (*p* = 0.026), age (*p* = 0.027), and the model overall (*p* = 0.035). In a post hoc analysis with follow-up split at 12 months, the HR for stage IV was 8.97 in the first 12 months and 3.94 thereafter (both *p* < 0.05), and age was not associated with mortality in either period. Delayed-entry models were fitted in the secondary cohort (*n* = 179; 172 patients with complete data and 50 deaths in the multivariable model), with time measured from diagnosis and patients entering the risk set at the start of systemic therapy. The proportional hazards assumption was met for all covariates and for the model overall (*p* = 0.66). Abbreviations: BRAF, B-Raf proto-oncogene serine/threonine kinase; CI, confidence interval; HR, hazard ratio; IPCW, inverse probability of censoring weighting.

**Table 5:** Overall survival, real-world progression-free survival, and time to next treatment or death by analysis anchor and treatment setting.

Outcome	*n*	Events, *n* (%)	Median, Months (95% CI)	24-Month Event-Free Probability (Kaplan-Meier) % (95% CI)	At Risk at 24 Months, *n*	*p*-Value
**From diagnosis** **(primary cohort,** ***n*** **= 165)**						
OS	165	49 (29.7)	66.0 (50.0–NE)	80.8 (73.5–86.3)	98	
**From the systemic therapy start** **(secondary cohort,** ***n*** **= 179)**						
OS	179	55 (30.7)	74.2 (36.0-NE)	69.2 (61.0–76.1)	57	
rwPFS	179	95 (53.1)	15.0 (10.9–25.9)	46.2 (38.2–53.9)	40	
TNT-D	179	93 (52.0)	19.9 (13.7–25.5)	47.2 (38.9–55.1)	41	
**By first-line systemic therapy intent** **(secondary cohort,** ***n*** **= 179)**						
OS, adjuvant	76	9 (11.8)	74.2 (74.2-NE)	91.5 (82.0–96.1)	25	<0.001
OS, neoadjuvant/perioperative	14	2 (14.3)	NR (14.4-NE)	NE	0	
OS, metastatic	89	44 (49.4)	27.7 (13.2-NE)	51.9 (40.5–62.1)	32	
rwPFS, adjuvant	76	28 (36.8)	NR (18.9-NE)	62.4 (49.5–72.9)	19	<0.001
rwPFS, neoadjuvant/perioperative	14	5 (35.7)	14.1 (5.1–14.1)	NE	0	
rwPFS, metastatic	89	62 (69.7)	8.4 (3.9–11.1)	33.0 (23.2–43.0)	21	
TNT-D, adjuvant	76	29 (38.2)	32.7 (19.9-NE)	60.4 (46.0–72.2)	18	<0.001
TNT-D, neoadjuvant/perioperative	14	5 (35.7)	12.4 (5.1–14.8)	NE	0	
TNT-D, metastatic	89	59 (66.3)	9.6 (5.6–14.3)	36.0 (25.9–46.2)	23	
**Sensitivity analysis: from diagnosis,** **full analytic cohort with delayed entry** **(** ***n*** **= 183)**						
OS	183	57 (31.1)	75.0 (66.0–119.5)	81.5 (74.4–86.8)	109	

Note. NR indicates that the median was not reached because the Kaplan–Meier estimate remained above 50% throughout the available follow-up. NE indicates not estimable; for medians, the confidence limit could not be calculated because the corresponding bound of the Kaplan–Meier confidence band did not fall below 50% within the available follow-up. For 24-month estimates in the neoadjuvant/perioperative group, no patients remained at risk at 24 months. In the sensitivity analysis, patients diagnosed before 2019 were counted as at risk only from 1 January 2019. Abbreviations: CI, confidence interval; NE, not estimable; NR, not reached; OS, overall survival; rwPFS, real-world progression-free survival; TNT-D, time to next treatment or death.

## Data Availability

The data presented in this study are available upon reasonable request from the corresponding author. The data are not publicly available due to patient privacy and ethical restrictions.

## Supplementary Materials

The following supporting information can be downloaded at: https://www.mdpi.com/article/10.3390/cancers18213540/s1, Checklist S1: CHEERS 2022 checklist; Table S1: Distribution of total direct medical costs by unit-cost source and cost category; Table S2: Cost per patient-month by phase of care and treatment period in the primary cohort (*n* = 165) with complete observed follow-up; Table S3: Sensitivity of 24-month mean cost estimates to alternative censoring specifications; Table S4: Duration, number of cycles, and reasons for ending first-line systemic therapy by treatment intent in the secondary cohort (*n* = 179). Reference [11] is cited in the Supplementary Materials.

## Abbreviations

The following abbreviations are used in this manuscript:

AJCC	American Joint Committee on Cancer
AUD	Australian dollar
BRAF	B-Raf proto-oncogene, serine/threonine kinase
CAD	Canadian dollar
CHEERS	Consolidated Health Economic Evaluation Reporting Standards
CI	Confidence interval
ctDNA	Circulating tumour DNA
CTLA-4	Cytotoxic T-lymphocyte-associated protein 4
EUR	Euro
GenAI	Generative artificial intelligence
HR	Hazard ratio
ICI	Immune checkpoint inhibitor
IPCW	Inverse probability of censoring weighting
IQR	Interquartile range
irAE	Immune-related adverse event
LAG-3	Lymphocyte-activation gene 3
MEK	Mitogen-activated protein kinase kinase
NE	Not estimable
NR	Not reached
OS	Overall survival
PD-1	Programmed cell death protein 1
RECIST	Response Evaluation Criteria in Solid Tumours
RMST	Restricted mean survival time
RSD	Serbian dinar
rwPFS	Real-world progression-free survival
SD	Standard deviation
TNM	Tumour, node, metastasis
TNT-D	Time to next treatment or death
USD	United States dollar
