# Supplementary material for: Real-World Direct Medical Costs and Clinical Outcomes of Immunotherapy and Targeted Therapy for Cutaneous Melanoma: A Bottom-Up Cost-of-Illness Study in a Public Healthcare System
Source: Cancers (Basel). 2026 Nov 4;18(21):3540. doi: 10.3390/cancers18213540 (PMC13762221; doi:10.3390/cancers18213540)
Supplement: Supplementary file 1 [file cancers-18-03540-s001.zip › Checklist_S1_CHEERS_2022 (1).pdf]

Checklist S1. CHEERS 2022 checklist.

Section/topic	Item no.	Guidance for reporting	Reported in
Title			
Title	1	Identify the study as an economic evaluation and specify the interventions being compared.	Title (cost-of-illness study; no interventions compared)
Abstract			
Abstract	2	Provide a structured summary that highlights context, key methods, results, and alternative analyses.	Abstract
Introduction			
Background and objectives	3	Give the context for the study, the study question, and its practical relevance for decision making in policy or practice.	Introduction
Methods			
Health economic analysis plan	4	Indicate whether a health economic analysis plan was developed and where available.	No health economic analysis plan was prepared
Study population	5	Describe characteristics of the study population (such as age range, demographics, socioeconomic, or clinical characteristics).	Section 2.2; Table 1
Setting and location	6	Provide relevant contextual information that may influence findings.	Introduction; Section 2.1
Comparators	7	Describe the interventions or strategies being compared and why chosen.	Not applicable: cost-of-illness study without compared interventions; treatments received are described in Section 2.4 and Table 1
Perspective	8	State the perspective(s) adopted by the study and why chosen.	Section 2.3
Time horizon	9	State the time horizon for the study and why appropriate.	Section 2.4; Table 3
Discount rate	10	Report the choice of discount rate(s) and reason chosen.	Section 2.3 (costs not discounted, as all costs had already been incurred)

Section/topic	Item no.	Guidance for reporting	Reported in
Selection of outcomes	11	Describe what outcomes were used as the measure(s) of benefit(s) and harm(s).	Section 2.4
Measurement of outcomes	12	Describe how outcomes used to capture benefit(s) and harm(s) were measured.	Sections 2.4 and 2.5
Valuation of outcomes	13	Describe the population and methods used to measure and value outcomes.	Not applicable: outcomes were reported in natural units and were not valued
Measurement and valuation of resources and costs	14	Describe how costs were valued.	Section 2.3; Supplementary Table S1
Currency, price date, and conversion	15	Report the dates of the estimated resource quantities and unit costs, plus the currency and year of conversion.	Section 2.3
Rationale and description of model	16	If modelling is used, describe in detail and why used. Report if the model is publicly available and where it can be accessed.	Not applicable: no decision-analytic model was used
Analytics and assumptions	17	Describe any methods for analysing or statistically transforming data, any extrapolation methods, and approaches for validating any model used.	Section 2.5
Characterising heterogeneity	18	Describe any methods used for estimating how the results of the study vary for subgroups.	Section 2.5; Tables 4 and 5; Figure 3
Characterising distributional effects	19	Describe how impacts are distributed across different individuals or adjustments made to reflect priority populations.	Not assessed
Characterising uncertainty	20	Describe methods to characterise any sources of uncertainty in the analysis.	Section 2.5
Approach to engagement with patients and others affected by the study	21	Describe any approaches to engage patients or service recipients, the general public, communities, or stakeholders (such as clinicians or payers) in the design of the study.	No patient, public, or stakeholder engagement in the study design
Results			

Section/topic	Item no.	Guidance for reporting	Reported in
Study parameters	22	Report all analytic inputs (such as values, ranges, references) including uncertainty or distributional assumptions.	Section 2.3; Supplementary Table S1
Summary of main results	23	Report the mean values for the main categories of costs and outcomes of interest and summarise them in the most appropriate overall measure.	Sections 3.2-3.5; Tables 2, 3, and 5; Figures 2 and 3; Supplementary Tables S2 and S4
Effect of uncertainty	24	Describe how uncertainty about analytic judgments, inputs, or projections affect findings. Report the effect of choice of discount rate and time horizon, if applicable.	Section 3.3; Table 3; Supplementary Table S3
Effect of engagement with patients and others affected by the study	25	Report on any difference patient/service recipient, general public, community, or stakeholder involvement made to the approach or findings of the study.	Not applicable: no engagement (see item 21)
Discussion			
Study findings, limitations, generalisability, and current knowledge	26	Report key findings, limitations, ethical or equity considerations not captured, and how these could affect patients, policy, or practice.	Section 4, including Strengths and limitations; Section 5
Other relevant information			
Source of funding	27	Describe how the study was funded and any role of the funder in the identification, design, conduct, and reporting of the analysis.	Funding; Conflicts of Interest
Conflicts of interest	28	Report authors conflicts of interest according to journal or International Committee of Medical Journal Editors requirements.	Conflicts of Interest

Note. The checklist follows Husereau et al. (2022). Items marked not applicable do not apply to a cost-of-illness study without compared interventions or a decision-analytic model. Abbreviations: CHEERS, Consolidated Health Economic Evaluation Reporting Standards.
