# Supplementary material for: Real-World Direct Medical Costs and Clinical Outcomes of Immunotherapy and Targeted Therapy for Cutaneous Melanoma: A Bottom-Up Cost-of-Illness Study in a Public Healthcare System
Source: Cancers (Basel). 2026 Nov 4;18(21):3540. doi: 10.3390/cancers18213540 (PMC13762221; doi:10.3390/cancers18213540)
Supplement: Supplementary file 1 [file cancers-18-03540-s001.zip › Supplementary Table S1.pdf]

Table S1. Distribution of total direct medical costs by unit-cost source and cost category.

Cost category	Total cost, RSD	Level 1: patient-specific invoice, %	Level 2: observed price from the cohort, %	Level 3: institutional tariff, %	Levels 1+2: prices observed in the payment system, %	Items, n
Immunotherapy and targeted therapy	2,454,455,140.76	28.20	56.60	15.20	84.80	5,676
Radiological and nuclear diagnostics	44,213,138.95	22.48	64.54	12.99	87.02	4,542
Inpatient and day-hospital care, other medications	31,912,635.51	39.73	1.33	58.94	41.06	25,573
Surgery and histopathology	18,834,981.25	43.46	40.22	16.32	83.69	5,546
Laboratory tests	10,374,128.58	42.43	0.94	56.62	43.38	13,930
Consultations	5,982,403.18	43.75	1.22	55.02	44.97	2,685
Molecular diagnostics	3,342,329.74	6.73	74.70	18.56	81.44	229
Tumour markers	2,829,255.82	56.99	1.61	41.40	58.60	2,287
TOTAL	2,571,944,013.79	28.45	55.54	16.00	84.00	60,468

Note. Data refer to the full analytic cohort (n = 183) over the entire observed follow-up period. The three-level unit-cost hierarchy was defined in the Methods: (1) the amount invoiced for the individual patient; (2) the amount invoiced for the same drug or procedure for another patient in the cohort, or obtained from the invoice-based drug price history; and (3) the institutional tariff. Levels 1 and 2 represent prices observed in the payment system. Percentages may not sum to exactly 100% due to rounding.

Abbreviations: RSD, Serbian dinar.
