# Supplementary material for: Real-World Direct Medical Costs and Clinical Outcomes of Immunotherapy and Targeted Therapy for Cutaneous Melanoma: A Bottom-Up Cost-of-Illness Study in a Public Healthcare System
Source: Cancers (Basel). 2026 Nov 4;18(21):3540. doi: 10.3390/cancers18213540 (PMC13762221; doi:10.3390/cancers18213540)
Supplement: Supplementary file 1 [file cancers-18-03540-s001.zip › Supplementary Table S2.pdf]

Table S2. Cost per patient-month by phase of care and treatment period in the primary cohort (n = 165) with complete observed follow-up.

Phase of care / treatment period	Patients, n	Total observed patient-months	Total cost, EUR	Cost per patient-month, EUR (95% CI)	Mean cost per patient, EUR [a]	Cost per patient-month ratio
Phases of care [b]						
Initial phase (first 6 months)	161	925.1	1,575,676	1,703 (1,362-2,078)	9,787	-
Continuing phase	148	3,914.1	14,959,601	3,822 (3,413-4,258)	101,078	-
Terminal phase (last 6 months before death)	49	287.1	1,651,497	5,753 (4,917-6,646)	33,704	-
Treatment periods [c]						
Before systemic therapy start	165	1,906.5	350,637	184 (149-229)	2,125	Ref.
During first-line systemic therapy	165	2,556.5	13,551,166	5,301 (4,789-5,875)	82,128	28.8
After first-line systemic therapy	56	663.3	4,284,971	6,460 (5,606-7,427)	76,517	35.1

Note. Values are unadjusted for censoring. Cost per patient-month was calculated as the total cost in each phase or period divided by the total observed patient-months, using all available follow-up. 95% CIs were obtained from 1,000 patient-level bootstrap resamples. Ratios are relative to the period before systemic therapy start.

[a] Mean cost per patient refers only to patients who entered each phase or period.

[b] Initial phase: first 6 months after diagnosis; terminal phase: last 6 months before death; continuing phase: the period in between. Overlaps between the initial and terminal phases were assigned to the terminal phase. Four patients who died within 6 months of diagnosis had no initial phase.

[c] During first-line systemic therapy: from its start until the start of the next line or death; after first-line systemic therapy: from the start of the second line onward.

Abbreviations: CI, confidence interval; EUR, euro.
