# Supplementary material for: Real-World Direct Medical Costs and Clinical Outcomes of Immunotherapy and Targeted Therapy for Cutaneous Melanoma: A Bottom-Up Cost-of-Illness Study in a Public Healthcare System
Source: Cancers (Basel). 2026 Nov 4;18(21):3540. doi: 10.3390/cancers18213540 (PMC13762221; doi:10.3390/cancers18213540)
Supplement: Supplementary file 1 [file cancers-18-03540-s001.zip › Supplementary Table S3.pdf]

Table S3. Sensitivity of 24-month mean cost estimates to alternative censoring specifications

A. Estimated mean cost per patient across censoring-adjustment approaches

**Estimator**	**Censoring-weight estimation**	**Anchor: date of diagnosis (n = 165)**		**Anchor: systemic therapy start (n = 179)**	
		24-month mean cost, EUR	95% CI	24-month mean cost, EUR	95% CI
Simple Bang-Tsiatis (base case)	Marginal (pooled across the anchor-specific cohort)	66,466	58,465-75,334	107,229	97,405-118,201
Simple Bang-Tsiatis	Stratified by calendar period of the anchor	71,537	62,583-81,227	111,341	98,149-124,627
Partitioned Bang-Tsiatis, 6-month intervals	Marginal (pooled across the anchor-specific cohort)	75,388	67,633-83,584	109,567	100,765-118,848
Partitioned Bang-Tsiatis, 6-month intervals	Stratified by calendar period of the anchor	77,901	69,952-86,876	112,734	102,801-123,377
Unadjusted sample mean (for reference only)	-	70,879	-	95,266	-
Relative range across IPCW specifications		+17.2%		+5.1%	

Notes. 95% confidence intervals were obtained from 1,000 patient-level bootstrap resamples, with censoring weights re-estimated in each resample.

Abbreviations: CI, confidence interval; IPCW, inverse probability of censoring weighting.

B. Distribution of patients, censoring, and costs by calendar period of the anchor date

Analysis anchor	Calendar-period stratum	Patients, n	Deaths during follow-up, n	Complete 24-month cost observations, n	Estimated probability of remaining uncensored through 24 months, $\hat{K}(24)$	Estimated 24-month mean cost per patient, EUR
Date of diagnosis	2019-2021	38	12	36	0.947	48,422
Date of diagnosis	2022-2023	74	26	70	0.934	70,536
Date of diagnosis	2024-2025	53	11	21	0.253	89,507
Date of diagnosis	All	165	49	127	0.735	66,466 (marginal); 71,537 (stratified)
Systemic therapy start	2019-2021	16	3	14	0.875	113,749
Systemic therapy start	2022-2023	66	29	61	0.906	104,074
Systemic therapy start	2024-2025	97	23	30	0.099	115,889
Systemic therapy start	All	179	55	105	0.460	107,229 (marginal); 111,341 (stratified)

Notes. Complete 24-month cost observations included patients followed for at least 24 months or those who died within 24 months. $\hat{K}(24)$ represents the estimated probability of remaining uncensored through 24 months, estimated using the Kaplan-Meier method, with censoring treated as the event.

C. Simulation-based diagnostic of the independent-censoring assumption

Quantity	Anchor from date of diagnosis	Anchor from systemic therapy start
Patients with complete 24-month cost observations, n	127	105
Reference mean 24-month cost per patient, EUR	65,671	95,187
Simulation replications, n	400	400
Relative bias of the simple estimator, %	+0.08%	-0.20%
Relative bias of the partitioned estimator, %	+0.11%	-0.14%
Median relative difference (partitioned vs simple), %	0.0%	0.0%
95% simulation range of the relative difference, %	-4.7% to +5.2%	-4.9% to +6.2%
Observed relative difference (partitioned vs simple), %	+13.4%	+2.2%
Simulations with a difference at least as large as observed, n/N (empirical p-value)	0 of 400 (p<0.003)	89 of 400 (p=0.22)
Interpretation	Evidence against independent censoring	No evidence against independent censoring

Notes. Simulations were restricted to patients with complete 24-month cost observations. In each of 400 replications, censoring times were drawn from the observed censoring distribution independently of accumulated costs during the follow-up period. The relative difference was calculated using the formula (partitioned estimate - simple estimate)/simple estimate × 100. Empirical one-sided p-values represent the proportion of simulations in which the difference was at least as large as the observed.
