# Supplementary material for: Real-World Direct Medical Costs and Clinical Outcomes of Immunotherapy and Targeted Therapy for Cutaneous Melanoma: A Bottom-Up Cost-of-Illness Study in a Public Healthcare System
Source: Cancers (Basel). 2026 Nov 4;18(21):3540. doi: 10.3390/cancers18213540 (PMC13762221; doi:10.3390/cancers18213540)
Supplement: Supplementary file 1 [file cancers-18-03540-s001.zip › Supplementary Table S4.pdf]

Table S4. Duration, number of cycles, and reasons for ending first-line systemic therapy by treatment intent in the secondary cohort (n = 179).

Characteristic	Adjuvant (n = 76)	Neoadjuvant/ perioperative (n = 14)	Metastatic (n = 89)	Total (n = 179)
On treatment at data cut-off, n	8	7	24	39
Duration of first-line therapy, months, median (IQR)[a]	11.1 (9.1-12.0)	11.5 (11.0-14.1)	6.8 (2.5-32.4)	10.8 (4.1-12.5)
Number of cycles, median (IQR)[b]	9.0 (8.0-11.0)	14.0 (5.3-15.5)	8.0 (4.0-25.5)	9.0 (5.0-14.0)
Completed first-line therapy, n	68	7	65	140
**Reason for ending, n (%)[c]**				
Completed as planned	43 (63.2)	3	0 (0.0)	46 (32.9)
Disease progression	14 (20.6)	2	42 (64.6)	58 (41.4)
Toxicity	7 (10.3)	0	4 (6.2)	11 (7.9)
Death	2 (2.9)	0	14 (21.5)	16 (11.4)
Other	2 (2.9)	2	5 (7.7)	9 (6.4)

Notes. [a]Kaplan-Meier estimates; patients on treatment at the data cut-off were censored. [b]Patients with a recorded number of cycles (n = 163). [c]Percentages of patients who completed first-line therapy; percentages are not shown for the neoadjuvant/perioperative group because of its small size. Other includes patient's wish, loss to follow-up, investigator decision, and unknown.
